# Associations of triglyceride-glucose index with N-terminal pro-B-type natriuretic peptide and mortality in middle-aged and elderly individuals

**DOI:** 10.3389/fendo.2025.1657724

**Published:** 2025-09-15

**Authors:** Haitao Xie, Le Shen, Jianghong Li, Chuxin Lv, Tong Sun, Peng Yu, Xiaohu Chen, Shuhua Tang

**Affiliations:** ^1^ First Clinical Medical College, Nanjing University of Chinese Medicine, Nanjing, China; ^2^ Cardiology Department, Jiangsu Province Hospital of Chinese Medicine, Nanjing, China; ^3^ Cardiology Department, Affiliated Hospital of Nanjing University of Chinese Medicine, Nanjing, China

**Keywords:** TyG index, elevated NT-proBNP, all-cause mortality, middle-aged and elderly, population study

## Abstract

**Background:**

The triglyceride-glucose (TYG) index is a simple marker for insulin resistance (IR). However, its relationship with elevated NT-proBNP levels is not well understood in middle-aged and elderly individuals without known cardiovascular diseases (CVD).

**Methods:**

The study cohort data were derived from National Health and Nutrition Examination Survey (NHANES) and inpatients of the Department of Cardiology at Jiangsu Provincial Hospital of Traditional Chinese Medicine (JSHTCM). Multivariable logistic regression was employed to assess the relationship between the TYG index and elevated NT-proBNP. Multivariable Cox proportional hazards models were used to estimate the adjusted risk ratio of the TYG index for all-cause mortality. Furthermore, restricted cubic spline (RCS) plots were generated to visually represent the linear or non-linear relationships between the TYG index and elevated NT-proBNP as well as all-cause mortality.

**Results:**

The age-standardized prevalence of elevated NT-proBNP among middle-aged and elderly individuals was 29.21% in females and 17.08% in males. A negative correlation was observed between the TYG index and elevated NT-proBNP, study cohort 1: [T3 vs T1: OR (95% CI): 0.73 (0.55, 0.96), *p* for trend= 0.027]; study cohort 2: [β (95% CI): -37.58 (-59.11, -16.06), *p* for trend=0.002]. Each unit increase in the TYG index is correlated with a 25% increase in the adjusted risk of all-cause mortality [HR (95% CI): 1.25 (1.08, 1.44), *p*=0.003]. The RCS plots supported the multivariate regression model findings.

**Conclusions:**

The TYG index level is negatively correlated with the incidence of elevated NT-proBNP and is associated with all-cause mortality, regardless of the presence of elevated NT-proBNP.

## Introduction

In recent years, the health burden caused by CVD has been increasing continually (1, 2), especially among middle-aged and elderly individuals, leading to the annual death of millions due to heart disease or stroke (3). This has become a significant public health challenge (4). N-terminal pro-B-type natriuretic peptide (NT-proBNP) is recognized as a significant cardiac biomarker, exhibiting a complex interplay with metabolic and cardiovascular health. Excessively elevated NT-proBNP levels typically indicate underlying cardiac pathology and adverse prognosis, whereas diminished levels may reflect insufficient protective natriuretic peptide signaling. Consequently, beyond its established utility in risk stratification and prognostic assessment of overt CVD such as heart failure (5–7), NT-proBNP may also serve as an indicator of subclinical cardiac stress or early myocardial dysfunction, particularly in individuals without a history of CVD, thereby providing crucial information for early diagnosis (8, 9).

IR is a common pathophysiological mechanism in the development of various metabolic diseases, and is also one of the main risk factors for CVD (10–12). The TYG index is a common alternative assessment indicator for IR (13–15), which is calculated according to fasting triglyceride and fasting blood glucose, with the advantages of being simple, easy to implement, and cost-effective. An increasing body of evidence supports the reliability of the TYG index in assessing IR (16–18). However, clinical observations have revealed a peculiar phenomenon where individuals with IR often exhibit “NT-proBNP deficiency” (19–21), which diverges somewhat from established understanding. Notably, prior investigations have predominantly focused on high-risk populations with diagnosed CVD. The potential association between the TYG index, a surrogate marker for IR, and circulating NT-proBNP levels in the general middle-aged and elderly individuals warrants further investigation.

In this research, we investigated whether elevated NT-proBNP levels are associated with the TYG index in individuals without known CVD. Moreover, we assessed the correlation between the TYG index and the risk of all-cause mortality across different populations.

## Methods

### Participants and study design

The study cohort 1 consisted of adult participants who participated in NHANES from 1999 to 2004, after excluding all individuals under 40 years old (n=4,064), BMI < 18.5 (n=157), a history of CVD (defined as self-reported coronary heart disease, heart attack, angina, stroke, or heart failure, n=10,371), missing data on a cardiac biomarker (NT-proBNP, n=9,920), missing data on TYG index and other covariates (n=382), substantial renal impairment (eGFR ≤15 ml/min/1.73 m², n=16). The final analytical population consisted of 6,216 participants.

**Figure 1:**
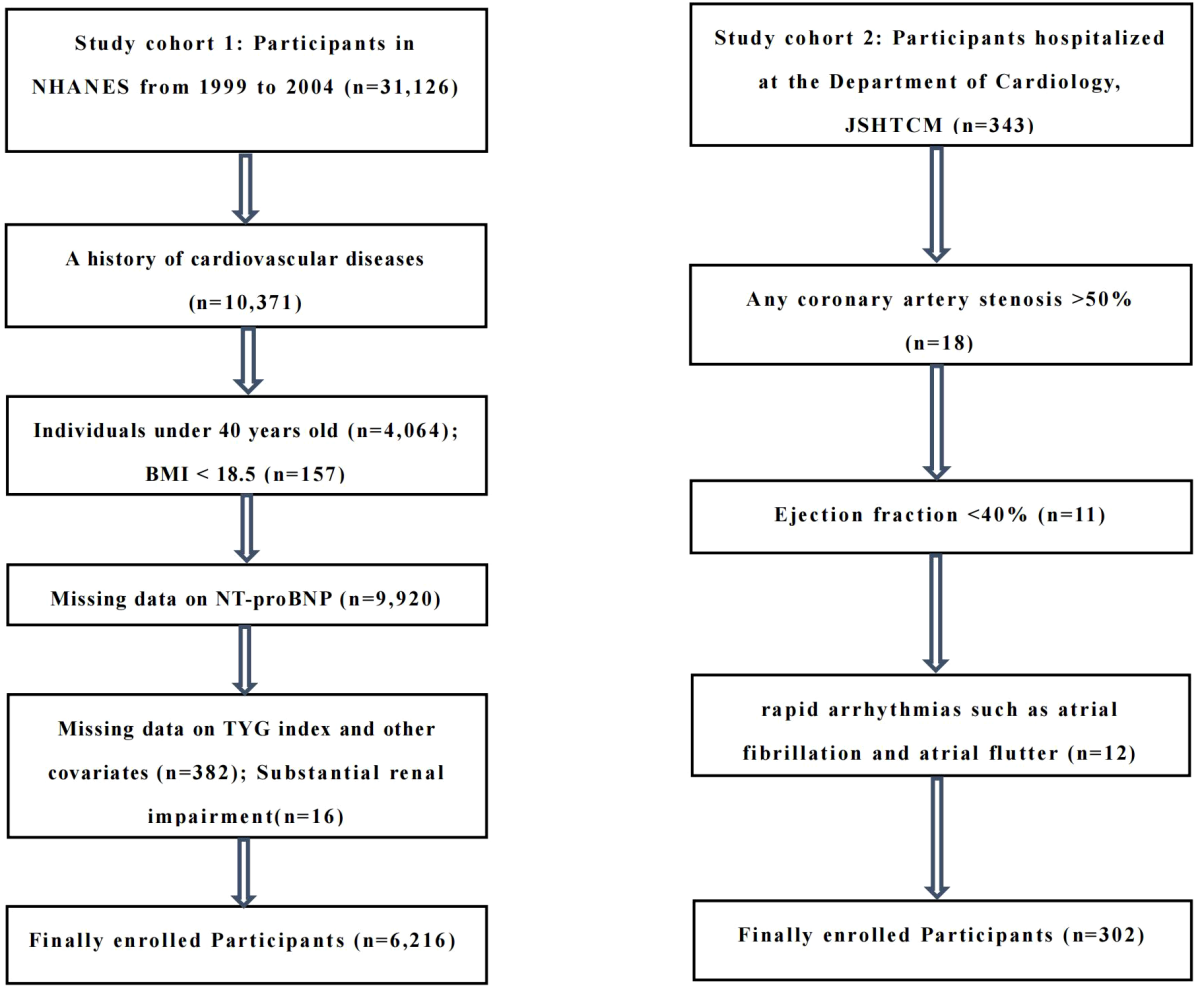
Study design and exclusion information flowchart.

The study cohort 2 included middle-aged and elderly patients, aged 40–79 years, who were hospitalized at the Department of Cardiology, JSHTCM, between December 2024 and June 2025. All subjects had no previous history of CVD and underwent routine electrocardiogram, echocardiography, and coronary CTA or coronary angiography during hospitalization. Exclusion criteria included: (1) any coronary artery stenosis >50% (n=18); (2) ejection fraction <40% (n=11); (3) rapid arrhythmias such as atrial fibrillation and atrial flutter (n=12). Ultimately, 302 middle-aged and elderly individuals were included in the analysis. The study design and exclusion details can be found in the flowchart (**Figure 1**).

### Assessment of TYG index

The TYG index was calculated as follows using these parameters as an exposure variable: Ln [triglycerides (mg/dl) * fasting glucose (mg/dl)/2] (22, 23). Fasting venous blood samples were collected from all participants after at least 8.5 hours of fasting. Serum triglycerides and glucose concentrations were measured with an automatic biochemical analyzer, and the specific levels were determined using coupling reaction enzyme methods and hexokinase reactions respectively.

### Elevated NT-proBNP

The study cohort 1 aimed to analyze NT-proBNP in stored serum samples, and the data are currently applicable to 1999-2004. The Roche Cobase 601 automated analyzer measured NT-proBNP levels in serum. The lower limit of detection was 5 pg/ml, and the upper limit was 35000 pg/ml. The coefficients of variation were 3.1% (low, 46 pg/ml) and 2.7% (high, 32805 pg/ml), respectively (24). Study cohort 2 was based on the measurement of serum NT-proBNP levels by the Laboratory Department of JSHTCM, with a low detection limit of 20 pg/mL. In the main analysis, we take reference of other research findings and stratified NT-proBNP levels based on established clinical reference ranges for cardiovascular biomarkers, we defined elevated NT-proBNP as NT-proBNP ≥125 pg/mL (25–28).

### All-cause mortality

In study cohort 1, from the start of the investigation (1999-2004), the follow-up period continued until December 31, 2019. Participant’s mortality data can be obtained by linking their personal identification code to the death certificate records in the National Death Index (NDI) based on the mortality files released by the National Center for Health Statistics (NCHS). Given the absence of endpoint event follow-up data within study cohort 2, the reporting of all-cause mortality outcomes is not applicable.

### Covariate definition

Participants in both study cohorts underwent detailed information collection to obtain sociodemographic data such as age, gender, Body Mass Index (BMI). Hypertension was defined by a mean systolic blood pressure of ≥140 mmHg, a diastolic blood pressure of ≥90 mmHg, self-reported physician-diagnosed hypertension, or the current use of antihypertensive medications. A detailed questionnaire was used to assess smoking status and categorize individuals as never, current, or former smokers. After fasting for 8.5 hours, participants provided a fasting venous blood sample, which was analyzed for total cholesterol (TC), high-density lipoprotein cholesterol (HDL-C), uric acid (UA), urea, creatinine, and glycated hemoglobin (HbA1c). Those with diabetes were defined as having a history of diabetes diagnosed by a physician or possessing an HbA1c of ≥6.5%. Renal function was evaluated using the Chronic Kidney Disease Epidemiology Collaboration (CKD-EPI) equation, and subjects exhibiting substantial renal impairment (eGFR ≤15 ml/min/1.73 m²) were omitted from the analysis. Medication use was obtained from the prescription records of medications used by participants in the past month, mainly including the following two categories: hypoglycemic and lipid-lowering drugs.

### Statistical analysis

**Figure 2:**
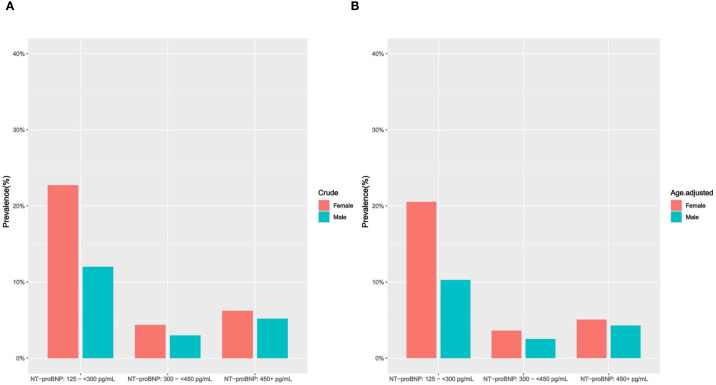
**(A, B)** Crude and age-adjusted prevalence of elevated NT-proBNP by gender (study cohort 1). **(A)** crude prevalence of elevated NT-proBNP (Female/Male); **(B)** age-adjusted prevalence of elevated NT-proBNP (Female/Male).

According to elevated NT-proBNP status, we assessed sociodemographic and cardiovascular risk factor characteristics among middle-aged and elderly individuals without known CVD. Normally distributed quantitative data are expressed as mean ± standard deviation (SD), and intergroup variations are evaluated using analysis of variance (ANOVA). For non-normally distributed data, the median (M) and interquartile range (IQR) are presented, and intergroup comparisons are performed using the Kruskal-Wallis rank-sum test. Categorical data are described using rates or proportions, and the chi-square test or Fisher’s exact test is applied to component comparisons. In the study cohort 1, we evaluated the crude prevalence of elevated NT-proBNP, then age-standardized the prevalence based on the age distribution of the U.S. adult population in 2000 (29), and visualized the data by histograms (**Figures 2A, B**).

In the study cohort 1, multivariable logistic regression and multivariable Cox proportional hazards models were used to evaluate the relationship between TYG index (exposure indicator) and elevated NT-proBNP levels and all-cause mortality risk, respectively. Leveraging prior research and accounting for potential confounders influencing exposure-outcome relationships (24–27), three different statistical inference models were used: Crude model: unadjusted; Model I: adjusted for age, gender, race, education level; Model II: further adjusted for BMI, hypertension, diabetes, smoking status, TC, HDL-C, urea, creatinine, UA, eGFR and use of hypoglycemic/lipid-lowering medications. Sensitivity analysis involved categorizing the TYG index into low (T1: 0-33.33%), medium (T2: 33.33%-66.67%), and high (T3:66.67%-100%) tertiles to test the robustness of the results, with the low TYG index (T1) set as a reference dummy variable to evaluate elevated circulating NT-proBNP and all-cause mortality risks in the tertiles, and the trend test was carried out. Moreover, we compared survival rates between the TYG index groups and subgroups using Kaplan-Meier curves, based on log-rank tests of survival rates.

Statistical inference model for the study cohort 2: Crude model: unadjusted; Model I: adjusted for age, gender; Model II: further adjusted for BMI, hypertension, diabetes, smoking, TC, HDL-C, urea, creatinine, UA, HbA1c, eGFR and use of hypoglycemic/lipid-lowering medications. The sensitivity analysis was consistent with study cohort 1.

In order to determine the potential nonlinear relationship between the TYG index and circulating levels of NT-proBNP and all-cause mortality risk, we employed a RCS model. This model facilitates the visual representation of the relationship between continuous variables and outcomes by segmenting the variable’s range into intervals through the placement of knots. To mitigate overfitting, we utilized the quantile method, establishing four knots at the 5th, 35th, 65th, and 95th percentiles of the data distribution, respectively. The model adjustment was consistent with the multivariable regression model.

In the subgroup analysis of the study cohort 1, data were stratified by gender (male/female), age (40-59y/≥ 60y), BMI (normal/overweight/obese), eGFR (< 60/60-< 90/≥ 90), hypertension (yes/no), diabetes (yes/no), and medications use (yes/no), with interaction tests conducted to assess consistency with the overall population results. These stratification factors are considered potential effect modifiers. Moreover, further analysis was conducted by stratifying elevated circulating NT-proBNP (yes/no) and age (40-59y/≥ 60y) to assess the association between TYG index and all-cause mortality in different subgroups.

In the study cohort 1, the recommended sampling survey weights were utilized in order to obtain unbiased results. R software (version 4.3.3; http://www.r-project.org) was used for conducting all statistical analyses. A two-tailed P-value < 0.05 was considered statistically significant.

### Methods consistency statement

We confirm that all analysis methods involving NHANES data in this study have been strictly adhered to in accordance with the NHANES survey methods and analytic guidelines recommended by the Centers for Disease Control and Prevention (CDC)/NCHS.

## Results

### Baseline characteristics of study participants

In the study cohort 1 (males vs females: 3,039 vs 3,177), the mean TYG index of the participants was 8.68 (0.67), with an average age of 55.2 years (54.8, 55.6). Examining the sociodemographic characteristics, it can be observed that individuals with a higher TYG index tend to be male, older in age, have less education, and are primarily non-Hispanic white. Regarding CVD risk factors, individuals with a higher TYG index frequently presented with comorbidities such as hypertension and diabetes, along with increased levels of BMI, smoking rates (former and current), serum urea, creatinine, uric acid, total cholesterol, triglycerides, fasting blood glucose, glycated hemoglobin, and mortality rate, while eGFR, HDL levels were relatively lower. Additionally, in the study cohort 2, apart from age and eGFR, the baseline characteristics of the participants remained largely consistent with those in the study cohort 1 (**Table 1**, **Supplementary Table S1**).

**Table 1:** Characteristics of US middle-aged and elderly individuals without known CVD by TYG index (study cohort 1).

Characteristic	All participants	Tertile 1	Tertile 2	Tertile 3	*P*
TYG index	8.68 (0.67)	6.922 - 8.403	8.403 - 8.931	8.931 - 13.246	
n	6,216	2,072	2,073	2,071	
Gender (%)					<0.001
Male	46.9	38.7	46.8	56.6	
Female	53.1	61.3	53.2	43.4	
Age	55.2 (54.8,55.6)	53.3 (52.7,54.0)	56.2 (55.4,57.1)	56.4 (55.9,57.0)	<0.001
Race (%)					<0.001
Mexican American	5.1	3.5	5.1	6.6	
Other Hispanic	5.0	3.1	6.3	5.8	
Non-Hispanic White	77.1	77.3	76.9	77.1	
Non-Hispanic Black	8.9	12.4	7.9	6.1	
Other Race	3.9	3.7	3.8	4.4	
Education (%)					<0.001
Less Than High School	18.9	15.3	19.2	22.6	
High School Diploma	26.1	22.8	28.6	27.3	
More Than High School	55.0	61.9	52.2	50.1	
Hypertension (%)					<0.001
No	58.7	67.7	56.4	50.6	
Yes	41.3	32.3	43.6	49.4	
Smoking (%)					0.023
Never	48.1	50.4	48.5	45.1	
Current	20.2	19.1	18.5	23.3	
Former	31.7	30.5	33	31.6	
BMI (kg/m^2^)	28.58 (5.98)	26.47 (5.36)	29.20 (6.25)	30.37 (5.62)	<0.001
Urea (mmol/l)	5.02 (1.76)	4.81 (1.66)	5.05 (1.73)	5.22 (1.87)	<0.001
Creatinine (mmol/l)	76.07 (33.39)	72.84 (23.44)	76.52 (35.96)	79.35 (39.51)	<0.001
eGFR (mL/min/1.73 m^2^)	76.07 (33.39)	91.62 (16.99)	88.34 (18.13)	87.73 (19.45)	<0.001
Uric acid (mmol/l)	320.42 (82.66)	288.56 (73.68)	325.54 (77.74)	352.06 (84.26)	<0.001
Total cholesterol (mmol/l)	5.48 (1.03)	5.15 (0.89)	5.49 (0.94)	5.87 (1.13)	<0.001
High-density lipoprotein (mmol/l)	1.38 (0.42)	1.61 (0.42)	1.36 (0.37)	1.14 (0.31)	<0.001
Triglycerides (mg/dl)	152.74 (153.39)	72.57 (18.22)	127.05 (23.25)	273.24 (228.52)	<0.001
Fasting blood glucose (mg/dl)	97.47 (30.52)	88.38 (10.05)	93.45 (15.49)	112.31 (47.95)	<0.001
Glycated hemoglobin (%)	5.59 (0.94)	5.31 (0.44)	5.49 (0.56)	6.02 (1.42)	<0.001
NT-proBNP (pg/mL)	130.2 (117.4,143.0)	127.9 (111.4,144.4)	144.1 (112.5,175.6)	118.2 (103.1,133.3)	0.361
Subclinical CVD (%)					0.054
No	78.1	76.4	77.5	80.6	
Yes	21.9	23.6	22.5	19.4	
Diabetes (%)					<0.001
No	91.4	97.3	94.0	81.9	
Yes	8.6	2.7	6.0	18.1	
Medications use (%)					0.706
No	93.1	92.9	93.5	93.1	
Yes	6.9	7.1	6.5	6.9	
Mortality rate (%)					<0.001
No	74.8	80.2	74.1	69.1	
Yes	25.2	19.8	25.9	30.9	

### The prevalence of elevated NT-proBNP

The crude prevalence of elevated NT-proBNP was 21.91% in study cohort 1, with a decline as the TYG index increased. Compared to middle-aged and elderly males, females exhibit a higher prevalence of elevated NT-proBNP (female vs male: 33.34% vs 20.2%). After further age standardization, the age-standardized prevalence rates of elevated circulating NT-proBNP are as follows: female: 29.21%, male: 17.08% (**Supplementary Table S3**, **Figures 2A, B**).

### Associations between the TYG index and elevated NT-proBNP

In the study cohort 1, when sociodemographic factors such as age, gender, race, and education level were adjusted, multivariate logistic regression models revealed that high TYG indexes were negatively associated with elevated NT-proBNP levels [OR (95% CI): 0.70 (0.61, 0.82), *p* < 0.001]. Upon further adjustment for model covariates (Model 2), the negative correlation between the two remained stable and still held significant statistical significance [OR (95% CI): 0.77 (0.64, 0.93), *p*=0.008]. Sensitivity analysis indicated that taking the low TYG index group (T1) as a reference, the risk of elevated NT-proBNP decreased by approximately 27% in the high TYG index group (T3) [OR (95% CI): 0.73 (0.55, 0.96), *p* for trend=0.027].

The results of study cohort 2 indicate that as the TYG index progressively increases, the serum NT-proBNP levels in middle-aged and elderly individuals show a declining trend, which is consistent with the multiple regression analysis of study cohort 1. Specifically, for each standard unit increase in the TYG index, the serum NT-proBNP level decreased by approximately 37.58 pg/mL [β (95% CI): -37.58 (-59.11, -16.06)]. Furthermore, the T3 group exhibited a more pronounced decrease in serum NT-proBNP levels than the T1 group (*p* for trend=0.002) **Table 2**, **Supplementary Table S2** shows detailed data.

**Table 2:** Adjusted associations of TYG index with elevated NT-proBNP (study cohort 1).

Outcome	Crude model		Model I		Model II	
	OR (95% CI)	*P* value	OR (95% CI)	*P* value	OR (95% CI)	*P* value
TYG	0.87 (0.78, 0.97)	0.016	0.70 (0.61, 0.82)	< 0.001	0.77 (0.64, 0.93)	0.008
TYG (tertile)						
T1	Reference	< 0.001	Reference	< 0.001	Reference	< 0.001
T2	1.06 (0.87, 1.29)	0.532	0.81 (0.66, 1.00)	0.058	0.91 (0.72, 1.17)	0.452
T3	0.82 (0.68, 1.01)	0.067	0.60 (0.48, 0.76)	< 0.001	0.73 (0.55, 0.96)	0.027
*P* for trend		0.077		< 0.001		0.027

Crude model: non-adjusted.

Model I: adjusted for age, gender, race, and education level.

Model II: adjusted for age, gender, race, education level, BMI, hypertension, diabetes, smoking status, TC, HDL-C, urea, creatinine, UA, eGFR and use of hypoglycemic/lipid-lowering medications.

**Figure 3:**
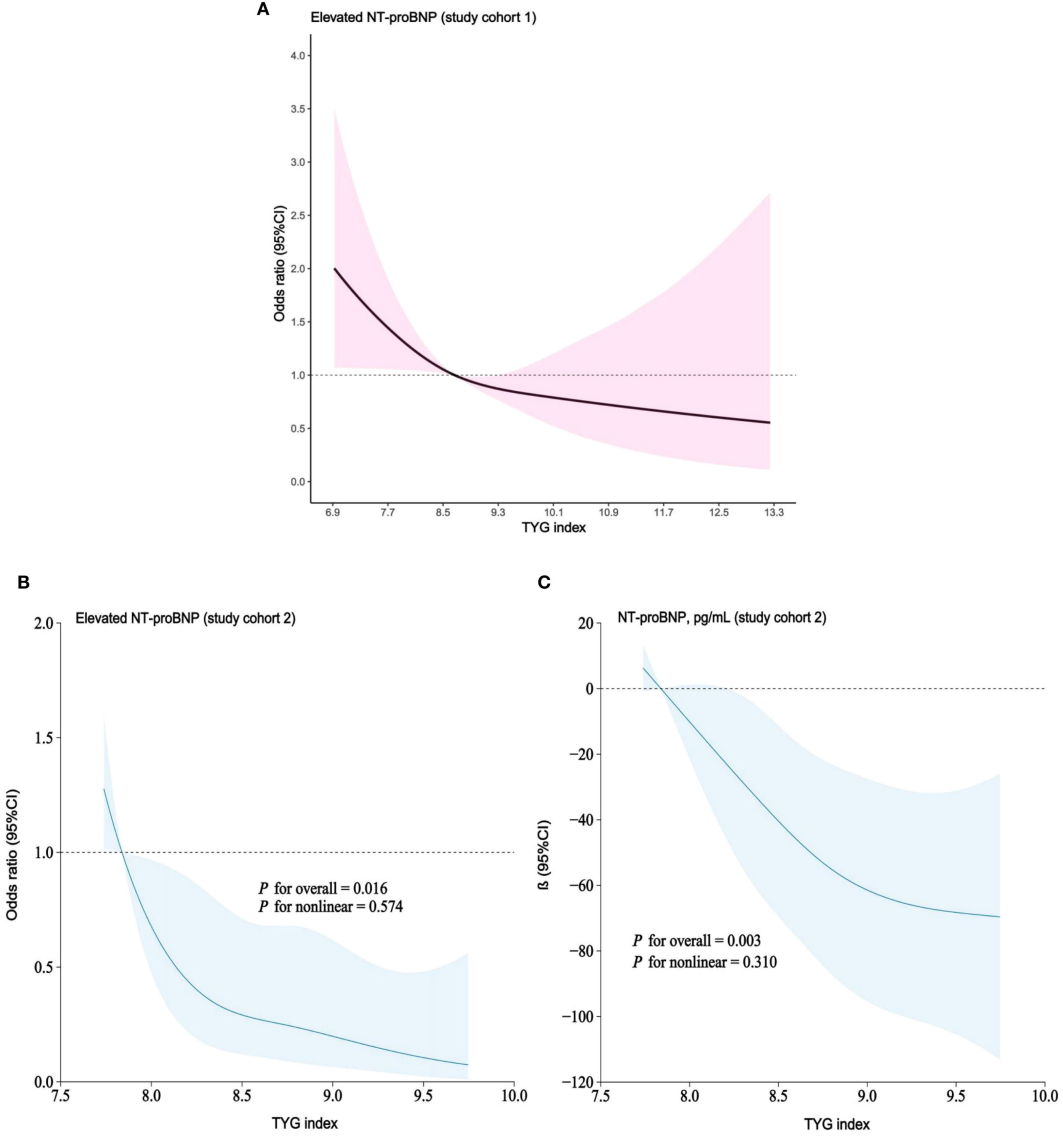
**(A–C)** RCS models demonstrating continuous association of TYG index with elevated NT-proBNP. **(A)** study cohort 1; RCS models adjusted for age, gender, race, education level, BMI, hypertension, diabetes, smoking status, TC, HDL-C, urea, creatinine, UA, eGFR and use of hypoglycemic/lipid-lowering medications. **(B, C)**. study cohort 2; RCS models adjusted for age, gender, BMI, hypertension, diabetes, smoking, TC, HDL-C, urea, creatinine, UA, HbA1c, eGFR and use of hypoglycemic/lipid-lowering medications.

In both study cohorts, the TYG index and elevated NT-proBNP were visualized on a continuous scale using RCS plots. The findings suggest that as the TYG index increases, the risk of elevated NT-proBNP gradually decreases, in line with the multivariable regression model (**Figures 3A-C**).

**Figure 4:**
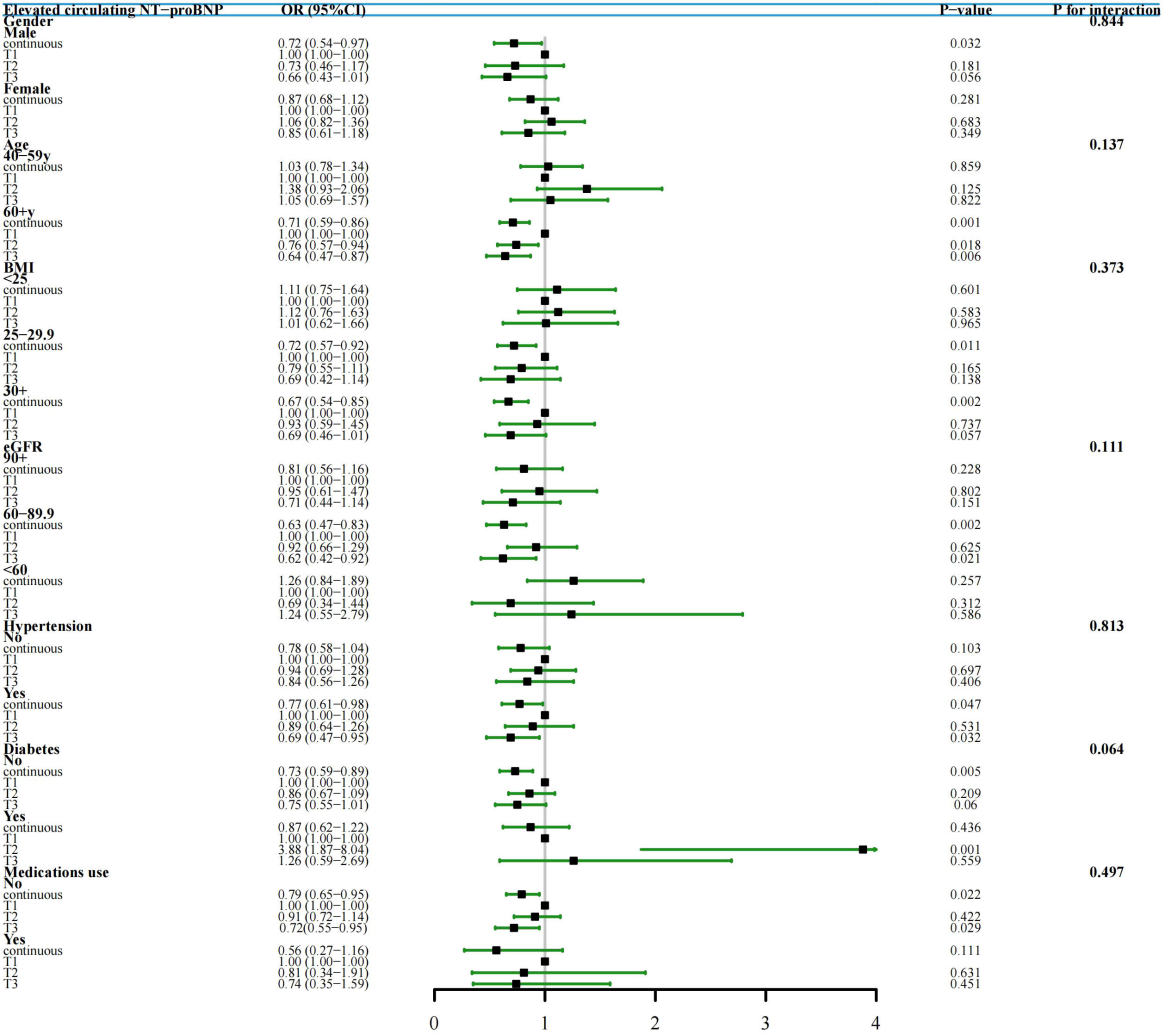
Subgroup Analysis and Forest Plots of TYG index with elevated NT-proBNP by gender, age, BMI, eGFR, hypertension, diabetes and medications use (study cohort 1). In various subgroup analyses, the adjustment for confounding factors, except for the stratification variables, remained consistent with Model II in the overall population.

Subgroup analyses were conducted in study cohort 1 to further validate the relationship between TYG index and elevated NT-proBNP across diverse populations stratified by gender, age, BMI, eGFR, hypertension, diabetes, and medications use. Our results indicate that individuals with a high TYG index group (T3) in different subgroups exhibit a lower risk of elevated NT-proBNP, and this negative correlation persists when the TYG index is continuous. Furthermore, interaction tests did not reveal any significant influence of gender, age, BMI, eGFR, hypertension, diabetes, and medications use on the association between TYG index and elevated NT-proBNP (**Figure 4**).

### Associations of TYG index with all-cause mortality

**Figure 5:**
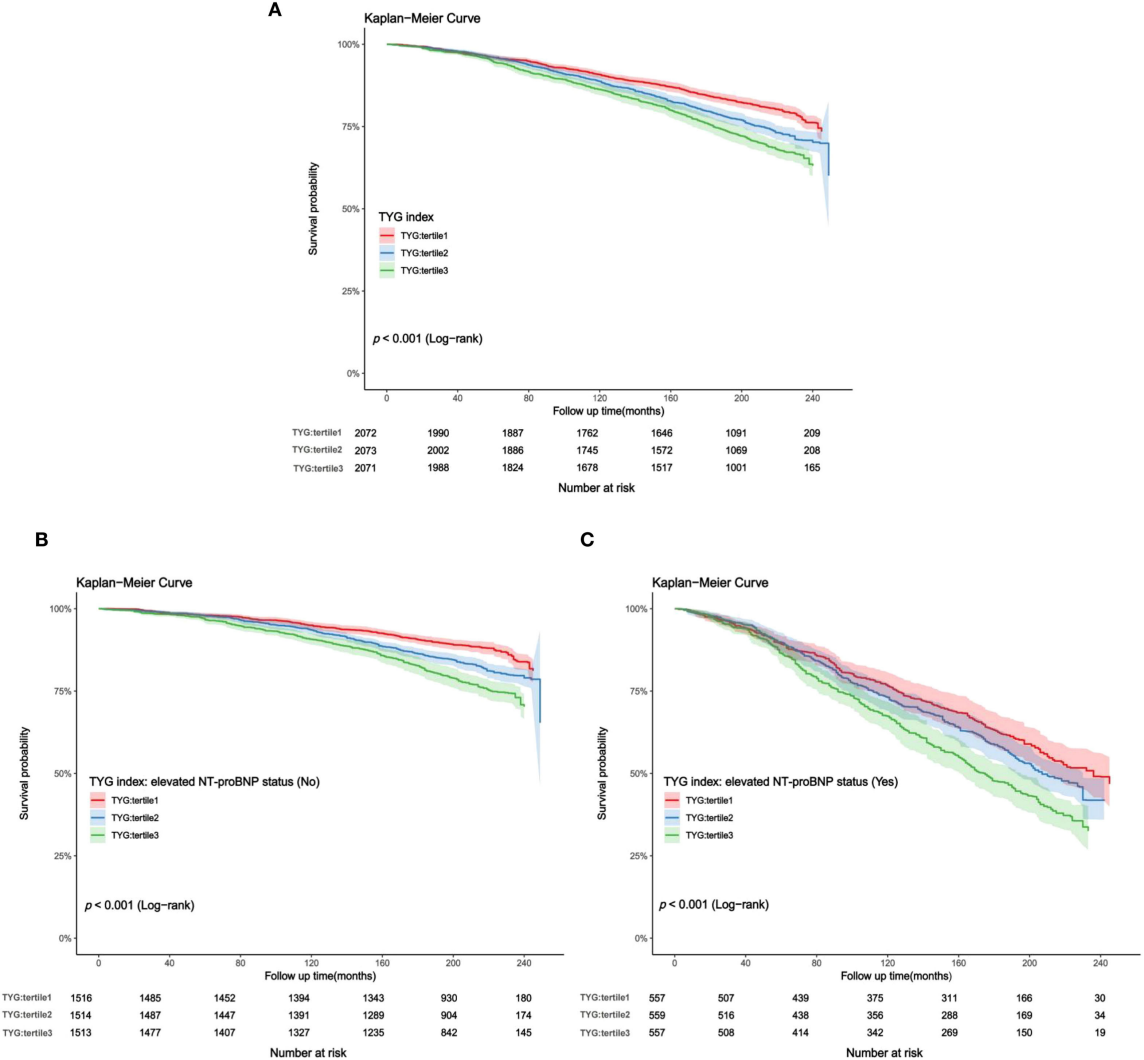
**(A–C)** Kaplan-Meier curves for TYG index and all-cause mortality (study cohort 1). **(A)** total middle-aged and elderly individuals; **(B)** middle-aged and elderly individuals without elevated NT-proBNP; **(C)** middle-aged and elderly individuals with elevated NT-proBNP.

In the study cohort 1, a total of 2,165 middle-aged and elderly individuals had died during the follow-up period as of December 31, 2019. The cumulative mortality rate in the non-elevated NT-proBNP group was 17.8%, compared to 51.8% in the elevated NT-proBNP group [absolute risk differences (ARD): 34%]. Kaplan-Meier survival curves showed that a higher TYG index was positively associated with an increased risk of all-cause mortality regardless of whether individuals had elevated NT-proBNP levels [survival probabilities: non-elevated NT-proBNP vs elevated NT-proBNP=82.2% vs 48.2%, (**Figures 5A-C**, all log-rank *p* value< 0.001)]. According to the multivariate Cox proportional hazards model, the adjusted all-cause mortality risk for the entire population increased by 25% for each unit increase in the TYG index [HR (95% CI): 1.25 (1.08, 1.44), *p*=0.003], with the risk ratio for those with elevated NT-proBNP at 1.12 (95% CI: 0.97, 1.29), while those with non-elevated NT-proBNP were 1.34 (95% CI: 1.13, 1.60). Sensitivity analysis suggests that compared to the lower TYG index group, the high TYG index group is associated with a 20% increased risk of adjusted all-cause mortality [HR (95% CI): 1.20 (1.02, 1.41), *p* for trend=0.031] (**Table 3**).

**Table 3:** Associations* (Hazard Ratio [95% CI]) between TYG index and all-cause mortality (study cohort 1).

Outcome	Crude model		Model I		Model II	
	HR (95% CI)	*P* value	HR (95% CI)	*P* value	HR (95% CI)	*P* value
TYG index	1.36 (1.25, 1.47)	< 0.001	1.25 (1.12, 1.39)	< 0.001	1.25 (1.08, 1.44)	0.003
TYG (tertile)						
T1	Reference	< 0.001	Reference	< 0.001	Reference	< 0.001
T2	1.34 (1.18, 1.52)	< 0.001	1.06 (0.95, 1.18)	0.316	1.05 (0.93, 1.18)	0.446
T3	1.66 (1.47, 1.88)	< 0.001	1.27 (1.11, 1.46)	< 0.001	1.20 (1.02, 1.41)	0.031
*P* for trend		< 0.001		< 0.001		0.028
Elevated NT-proBNP status (No)						
TYG index	HR (95% CI)	*p* value	HR (95% CI)	*p* value	HR (95% CI)	*p* value
TYG (tertile)	1.46 (1.31, 1.62)	< 0.001	1.30 (1.15, 1.47)	< 0.001	1.34 (1.13, 1.60)	0.001
T1	Reference	< 0.001	Reference	< 0.001	Reference	< 0.001
T2	1.46 (1.25, 1.70)	< 0.001	1.11 (0.96, 1.28)	0.178	1.15 (0.97, 1.38)	0.112
T3	2.01 (1.68, 2.39)	< 0.001	1.43 (1.20, 1.69)	< 0.001	1.44 (1.16, 1.80)	0.001
*P* for trend		< 0.001		< 0.001		< 0.001
Elevated NT-proBNP (Yes)						
TYG index	HR (95% CI)	*p* value	HR (95% CI)	*p* value	HR (95% CI)	*p* value
TYG (tertile)	1.44 (1.25, 1.65)	< 0.001	1.28 (1.13, 1.45)	< 0.001	1.12 (0.97, 1.29)	0.139
T1	Reference	< 0.001	Reference	< 0.001	Reference	< 0.001
T2	1.18 (0.95, 1.47)	0.133	1.03 (0.85, 1.26)	0.741	0.95 (0.78, 1.16)	0.627
T3	1.56 (1.24, 1.95)	< 0.001	1.23 (1.00, 1.51)	0.049	0.99 (0.78, 1.27)	0.958
*P* for trend		< 0.001		0.048		0.962

Crude model: non-adjusted.

Model I: adjusted for age, gender, race, and education level.

Model II: adjusted for age, gender, race, education level, BMI, hypertension, diabetes, smoking status, TC, HDL-C, urea, creatinine, UA, eGFR and use of hypoglycemic/lipid-lowering medications.

In the study cohort 1, the RCS curves adjusted for multiple variables indicate that with the gradual increase in the TYG index, the overall population experiences a gradual rise in all-cause mortality risk. This trend is consistent in both the non-elevated circulating NT-proBNP group and the elevated circulating NT-proBNP group, mirroring the findings observed in the overall population (**Supplementary Figures S1A-C**). Furthermore, the subgroup analysis results also support these findings (**Supplementary Table S4**, **Supplementary Figure S2-S5**).

## Discussion

In both study cohorts, we analyzed the relationship between the TYG index and the risk of elevated circulating NT-proBNP and all-cause mortality in middle-aged and elderly individuals without known CVD. A negative relationship was found between the TYG index and circulating NT-proBNP levels, which implies that higher IR levels are associated with lower NT-proBNP levels. As well, the TYG index demonstrated a positive association with elevated all-cause mortality across all participants, irrespective of the presence of elevated circulating NT-proBNP. These findings indicate that in general middle-aged and elderly individuals without known CVD, IR may still pose a certain risk burden for long-term death outcomes.

IR is commonly attributed to impairments in glucose metabolism within tissues mediated by insulin, serving as a significant pathological basis for underlying metabolic disorders like diabetes and obesity (30). Hyperinsulinemic euglycemic clamp testing is currently the gold standard for assessing IR. However, its invasive nature and substantial cost render it impractical for widespread clinical use. As one of the alternative indexes to evaluate IR, the TYG index offers a practical and broadly applicable substitute (31–33), and numerous studies have validated its potential and dependability on forecasting IR. As demonstrated in Iwakura et al. (34), individuals with preserved ejection fraction with heart failure can use the TYG index as a novel indicator of IR. Furthermore, Brito and colleagues (35) highlighted the notable efficacy of the TYG index in predicting IR in adolescent populations. Therefore, the use of the TYG index to evaluate IR has significant advantages in clinical practice.

NT-proBNP has been consistently recognized as a cardiac biomarker in CVD and is widely employed for the early detection, diagnosis, and clinical management of adverse cardiovascular events such as heart failure following a myocardial infarction. Our findings indicated that the level of NT-proBNP decreased with the increase of the TYG index, suggesting a potential correlation where a higher TYG index could be associated with lower circulating NT-proBNP. In the past, only few studies have explored the relationship between TYG index and NT-proBNP, mainly in CVD cohorts. Olsen et al. (36) revealed that NT-proBNP levels in patients with metabolic syndrome were low and negatively correlated with blood lipids and insulin. A survey was performed by Wang et al. (37) on a substantial sample size further corroborated the diminished plasma BNP levels in overweight and obese subjects compared to those with a normal BMI. Furthermore, Jujić et al. (38) reported that high levels of atrial natriuretic peptide within the normal range among middle-aged individuals are linked to a reduced risk of IR. In this study, based on NT-proBNP levels, we examined the relationship between elevated circulating NT-proBNP and the TYG index in general middle-aged and elderly individuals. These findings are basically consistent with the above findings and complement and expand on the previous research results.

From the perspective of molecular biological mechanisms, NT-proBNP is a synthetic product of ventricular myocytes, typically in response to physiological signals such as ventricular wall stretching, sodium levels or changes in systemic blood pressure. In the study analysis, considering the potential impact of obesity on NT-proBNP (39), we controlled the BMI factor and excluded individuals with low body weight. Prior observational investigations have found a deficiency of NT-proBNP in obese cohorts (40, 41), alongside upregulated expression of NT-proBNP clearance receptors within subcutaneous adipose tissue under hyperinsulinemic conditions (42, 43). This phenomenon is postulated to be a consequence of enhanced natriuretic peptide clearance by adipose tissue, which to some extent establishes a mechanistic link between obesity, IR, and diminished circulating NT-proBNP levels. Within diabetes prevention cohorts, studies have revealed that NT-proBNP levels more accurately reflect an individual’s insulin sensitivity (44); specifically, lower circulating NT-proBNP is associated with reduced insulin sensitivity, a relationship that persists independent even after adjusting for obesity metrics, mirroring findings from cross-sectional studies (39). Bachmann et al. (45) reported a significant reduction in N-terminal proatrial natriuretic peptide (NT-proANP) following insulin infusion, which concomitantly stimulated and upregulated the expression of NT-proANP clearance receptors in adipose tissue, thus promoting the clearance of circulating natriuretic peptides. We hypothesize that a similar mechanism may contribute to the observed reduction in NT-proBNP levels. Therefore, it may be reasonable to observe a lower prevalence of elevated circulating NT-proBNP (i.e., lower NT-proBNP levels) in individuals with a higher TYG index. However, further investigation is warranted to fully elucidate the specific biological mechanisms underpinning the relationship between IR and NT-proBNP.

Researchers have previously reported an association between the TYG index and mortality risks from all causes and cardiovascular disease (46–49). As Li et al. (50) noted, TYG index and all-cause mortality have a U-shaped relationship in CVD patients. In individuals with diabetes or prediabetes, researchers have observed that the TYG index can serve as a reliable predictor of all-cause and cardiovascular mortality, with higher predictive accuracy than other indicators (51). Chen et al. (52) reported differences in the TYG index and mortality risk across different age groups in the general population, especially more significant in non-elderly individuals. In addition, a study from the MIMIC database concluded that the TYG index can be effectively used for predictive purposes in patients with cerebrovascular diseases (53). Therefore, the TYG index can be considered a critical indicator for assessing the health status of individuals or groups, monitoring the adverse events, and taking targeted measures to manage health.

As we found in this study, there is a positive correlation between the TYG index and all-cause mortality among middle-aged and elderly individuals with no CVD history. In individuals with elevated NT-proBNP levels or non-elevated NT-proBNP levels, mortality risks are similar to those in the overall population. Additionally, the relationship remains stable even after adequate adjustment, and subgroup analysis results from different populations also support these findings. As a simple and alternative indicator of IR, the association between the TYG index and all-cause mortality may be explained by the following mechanisms: 1. Prolonged high insulin levels caused by IR and tissue glucose metabolism disorders can lead to systemic metabolic dysfunction, including metabolic disorders associated with fatty liver disease and type 2 diabetes as well as increased mortality associated with diabetes over the long term; 2. It has been proven that high levels of the TYG index are associated with an increased risk of developing cancers like colorectal and breast (54, 55), which results in reduced survival rates for the individual; 3. The TYG index may be linked to a higher risk of obesity, where obese individuals may experience nutrient excess, raising the risk of developing other chronic diseases and subsequently increasing overall mortality rates.

Our study holds several significant clinical implications. In middle-aged and elderly individuals without known CVD, the association between elevated circulating NT-proBNP and the TYG index may provide new insights into individual IR levels, support the early identification of individual IR levels through NT-proBNP as a cardiac biomarker, and provide recommendations for the formulation of clinical strategy. This study indicates that the association between the TYG index and all-cause mortality expands the range of potential risk populations, highlighting the importance of looking at long-term adverse outcome risks associated with the TYG index not only limited to high-risk cohorts such as diabetes and CVD, but also in general middle-aged and elderly individuals. The purpose of the present study was to determine for the first time whether the TYG index correlated with elevated circulating NT-proBNP as well as all-cause mortality among middle-aged and elderly individuals without known CVD. We combined data from two study cohorts, and adjusted for various potential confounding factors, which undoubtedly constitute a significant strength of this study. Future research should further investigate the possible key biological mechanisms that may exist between IR and elevated circulating NT-proBNP, to provide empathetic evidence to support our findings.

It is important to acknowledge our study’s limitations. First, as an observational study, we cannot determine the causality we have found and establish the temporality of the associations. Second, since study cohort 2 was derived from hospitalized patients and lacked endpoint event data, it was not possible to revalidate the relationship between the TYG index and mortality risk in study cohort 2, and compared to coronary angiography, coronary CTA has a slightly lower diagnostic sensitivity, which may potentially lead to information bias regarding the personal history of CVD. Furthermore, circulating NT-proBNP levels are susceptible to the direct influence of subclinical left ventricular dysfunction. However, the absence of echocardiographic data and left ventricular function assessments in study cohort 1 may have, to some extent, potentially influenced the association between the TYG index and NT-proBNP. Finally, although we established a multivariate regression model to reduce confounding factors interference, we cannot rule out the possibility of residual confounding factors.

## Conclusion

In middle-aged and elderly individuals without known CVD, the TYG index demonstrates an inverse relationship with the onset of elevated circulating NT-proBNP levels and is associated with all-cause mortality in the general population. We advocate the TYG index as a valuable risk indicator, which holds positive significance for early risk identification and prognosis assessment.

## Data Availability

Publicly available datasets were analyzed in this study. This data can be found here: https://wwwn.cdc.gov/nchs/nhanes/Default.aspx.
