# Supplementary material for: Associations of triglyceride-glucose index with N-terminal pro-B-type natriuretic peptide and mortality in middle-aged and elderly individuals
Source: Front Endocrinol (Lausanne). 2025 Sep 15;16:1657724. doi: 10.3389/fendo.2025.1657724 (PMC12444021; doi:10.3389/fendo.2025.1657724)
Supplement: Supplementary file 1 [file DataSheet1.docx]

**Associations of triglyceride-glucose index with N-Terminal Pro-B-Type Natriuretic Peptide and mortality in middle-aged and elderly individuals**

**Contact information:**

Haitao Xie: xht18356003693@163.com

Le Shen: leshen1024@126.com

Jianghong Li: lijianghong2022@163.com

Chuxin Lv: Lvchuxin0113@126.com

Tong Sun: suntong@163.com

Peng Yu: yupengdoctor@126.com

Xiaohu Chen: chenxhdoctor@126.com

Shuhua Tang: [suewang10@163.com](mailto:suewang10@163.com)

Table S1. Characteristics of middle-aged and elderly individuals without known CVD by TYG index, department of Cardiology, JSHTCM (study cohort 2).

|  | Overall | Tertile 1 | Tertile 2 | Tertile 3 | *p* |
| --- | --- | --- | --- | --- | --- |
| TYG index | 8.625 (0.548) | 7.392-8.372 | 8.372-8.804 | 8.804-10.563 |  |
| n | 302 | 101 | 100 | 101 |  |
| Gender (%) |  |  |  |  | 0.335 |
| Male | 43.7 | 39.6 | 42.0 | 49.5 |  |
| Female | 56.3 | 60.4 | 58.0 | 50.5 |  |
| Age | 62.8 ± 9.1 | 64.9 ± 8.6 | 63.3 ± 9.2 | 60.0 ± 8.9 | <0.001 |
| Hypertension (%) |  |  |  |  | 0.209 |
| No | 32.12 | 37.62 | 26.00 | 32.67 |  |
| Yes | 67.88 | 62.38 | 74.00 | 67.33 |  |
| Smoking (%) |  |  |  |  | 0.043 |
| Never | 76.82 | 83.17 | 79.00 | 68.32 |  |
| Current | 16.56 | 10.89 | 13.00 | 25.74 |  |
| Former | 6.62 | 5.94 | 8.00 | 5.94 |  |
| BMI (kg/m^2^) | 25.19 (3.57) | 23.94 (3.61) | 25.67 (3.09) | 25.97 (3.66) | <0.001 |
| Urea (mmol/l) | 5.62 (1.38) | 5.60 (1.28) | 5.53 (1.30) | 5.72 (1.55) | 0.548 |
| Creatinine (mmol/l) | 69.11 (17.48) | 68.45 (17.38) | 68.60 (17.15) | 70.26 (18.00) | 0.676 |
| eGFR (mL/min/1.73 m^2^) | 89.25 (14.55) | 87.92 (14.44) | 88.89 (15.20) | 90.94 (13.95) | 0.005 |
| Uric acid (mmol/l) | 319.06 (87.37) | 305.73 (79.36) | 312.75 (90.23) | 338.64 (89.52) | 0.01 |
| Total cholesterol (mmol/l) | 4.31 (1.09) | 4.15 (0.99) | 4.27 (1.08) | 4.50 (1.17) | 0.043 |
| High-density lipoprotein (mmol/l) | 1.19 (0.29) | 1.30 (0.32) | 1.16 (0.27) | 1.10 (0.24) | <0.001 |
| Triglycerides (mg/dl) | 131.66 (78.58) | 75.38 (17.30) | 110.08 (20.09) | 209.30 (90.20) | <0.001 |
| Fasting blood glucose (mg/dl) | 98.45 (20.55) | 87.81 (12.15) | 99.45 (19.32) | 108.09 (23.35) | <0.001 |
| Glycated hemoglobin (%) | 6.14 (0.90) | 5.81 (0.50) | 6.21 (0.92) | 6.40 (1.08) | <0.001 |
| NT-proBNP (pg/mL) | 85.40 (90.87) | 110.25 (106.04) | 91.70 (96.95) | 54.29 (51.65) | <0.001 |
| Diabetes (%) |  |  |  |  | <0.001 |
| No | 78.48 | 93.07 | 78.00 | 64.36 |  |
| Yes | 21.52 | 6.93 | 22.00 | 35.64 |  |
| Medications use (%) |  |  |  |  | 0.808 |
| No | 53.97 | 54.46 | 56.00 | 51.49 |  |
| Yes | 46.03 | 45.54 | 44.00 | 48.51 |  |

Table S2. Adjusted associations of TYG index with NT-proBNP, department of Cardiology, JSHTCM (study cohort 2).

Crude model: non-adjusted;

Model I: adjusted for age, gender;

Model II: adjusted for age, gender, BMI, hypertension, diabetes, smoking, TC, HDL-C, urea, creatinine, UA, HbA1c, eGFR and use of hypoglycemic/lipid-lowering medications.

| Outcome | crude model |  | model I |  | model II |  |
| --- | --- | --- | --- | --- | --- | --- |
|  | OR (95% CI) | *p* value | OR (95% CI) | *p* value | OR (95% CI) | *p* value |
| TYG | 0.32 (0.17, 0.59) | < 0.001 | 0.38 (0.19, 0.72) | < 0.001 | 0.45 (0.26, 0.85) | 0.003 |
| TYG (tertile) |  |  |  |  |  |  |
| T1 | Reference |  | Reference |  | Reference |  |
| T2 | 0.74 (0.39, 1.40) | 0,348 | 0.78 (0.39, 1.54) | 0.469 | 0.78 (0.36, 1.68) | 0.523 |
| T3 | 0.26 (0.12, 0.57) | < 0.001 | 0.36 (0..15, 0.83) | 0.017 | 0.51 (0.28, 0.77) | 0.014 |
| *P* for trend |  | < 0.001 |  | < 0.019 |  | 0.018 |
| Outcome | crude model |  | model I |  | model II |  |
|  | β (95% CI) | *p* value | β (95% CI) | *p* value | β (95% CI) | *p* value |
| TYG | -43.71 (-61.80, -25.61) | < 0.001 | -32.11 (-49.61, -14.61) | < 0.001 | -37.58 (-59.11, -16.06) | < 0.001 |
| TYG (tertile) |  |  |  |  |  |  |
| T1 | Reference |  | Reference |  | Reference |  |
| T2 | -18.55 (-42.91, 5.82) | 0.136 | -13.61 (-36.72, 9.49) | 0.249 | -15.72 (-40.53, 9.08) | 0.215 |
| T3 | -55.96 (-80.26, -31.66) | < 0.001 | -39.79 (-63.37, -16.21) | 0.001 | -43.38 (-70.88, -15.88) | 0.002 |
| *P* for trend |  | < 0.001 |  | 0.001 |  | 0.002 |

Table S3. Crude and age-adjusted prevalence of elevated NT-proBNP by gender (study cohort 1).

| Elevated circulating NT-proBNP | Male | Female |
| --- | --- | --- |
| Crude rate (%) |  |  |
| 125 - <300 pg/mL | 12.01% | 22.73% |
| 300 - <450 pg/mL | 2.99% | 4.38% |
| ≥450 pg/mL | 5.20% | 6.23% |
| Age-ajusted rate (%) |  |  |
| 125 - <300 pg/mL | 10.28% | 20.53% |
| 300 - <450 pg/mL | 2.52% | 3.62% |
| ≥450 pg/mL | 4.28% | 5.06% |

Table S4. Associations* (Hazard Ratio [95% CI]) between TYG index and all-cause mortality (study cohort 1).

| Outcome | crude model |  | model I |  | model II |  |
| --- | --- | --- | --- | --- | --- | --- |
| Age: 40−59 year | | | | | | |
|  | HR (95% CI) | *p* value | HR (95% CI) | *p* value | HR (95% CI) | *p* value |
| TYG index | 1.56 (1.33, 1.82) | < 0.001 | 1.44 (1.18, 1.76) | < 0.001 | 1.49 (1.14, 1.93) | 0.003 |
| TYG (tertile) |  |  |  |  |  |  |
| T1 | Reference | < 0.001 | Reference | < 0.001 | Reference | < 0.001 |
| T2 | 1.21 (0.87, 1.68) | 0.27 | 1.05 (0.75, 1.48) | 0.784 | 1.11 (0.74, 1.63) | 0.655 |
| T3 | 1.93 (1.45, 2.57) | < 0.001 | 1.54 (1.13, 2.11) | 0.007 | 1.46 (1.05, 2.08) | 0.028 |
| *P* for trend |  | < 0.001 |  | 0.008 |  | 0.024 |
| Age: 40−59 year and elevated NT-proBNP status (No) | | | | | | |
| TYG index | HR (95% CI) | *p* value | HR (95% CI) | *p* value | HR (95% CI) | *p* value |
| TYG (tertile) | 1.58 (1.32, 1.88 ) | < 0.001 | 1.46 (1.18, 1.80) | < 0.001 | 1.53 (1.19, 1.96) | < 0.001 |
| T1 | Reference | < 0.001 | Reference | < 0.001 | Reference | < 0.001 |
| T2 | 1.21 (0.87, 1.69) | 0.263 | 1.06 (0.75, 1.49) | 0.758 | 1.16 (0.77, 1.76) | 0.478 |
| T3 | 2.04 (1.52, 2.74) | < 0.001 | 1.62 (1.19, 2.23) | 0.002 | 1.59 (1.10, 2.31) | 0.013 |
| *P* for trend |  | < 0.001 |  | 0.004 |  | 0.009 |

Crude model: non-adjusted;

Model I: adjusted for age, gender, race, and education level;

Model II: adjusted for age, gender, race, education level, BMI, hypertension, diabetes, smoking status, TC, HDL-C, urea, creatinine, UA, eGFR and use of hypoglycemic/lipid-lowering medications.

Figure S1(A-C). RCS models demonstrating continuous associations of TYG index with all-cause mortality (study cohort 1).


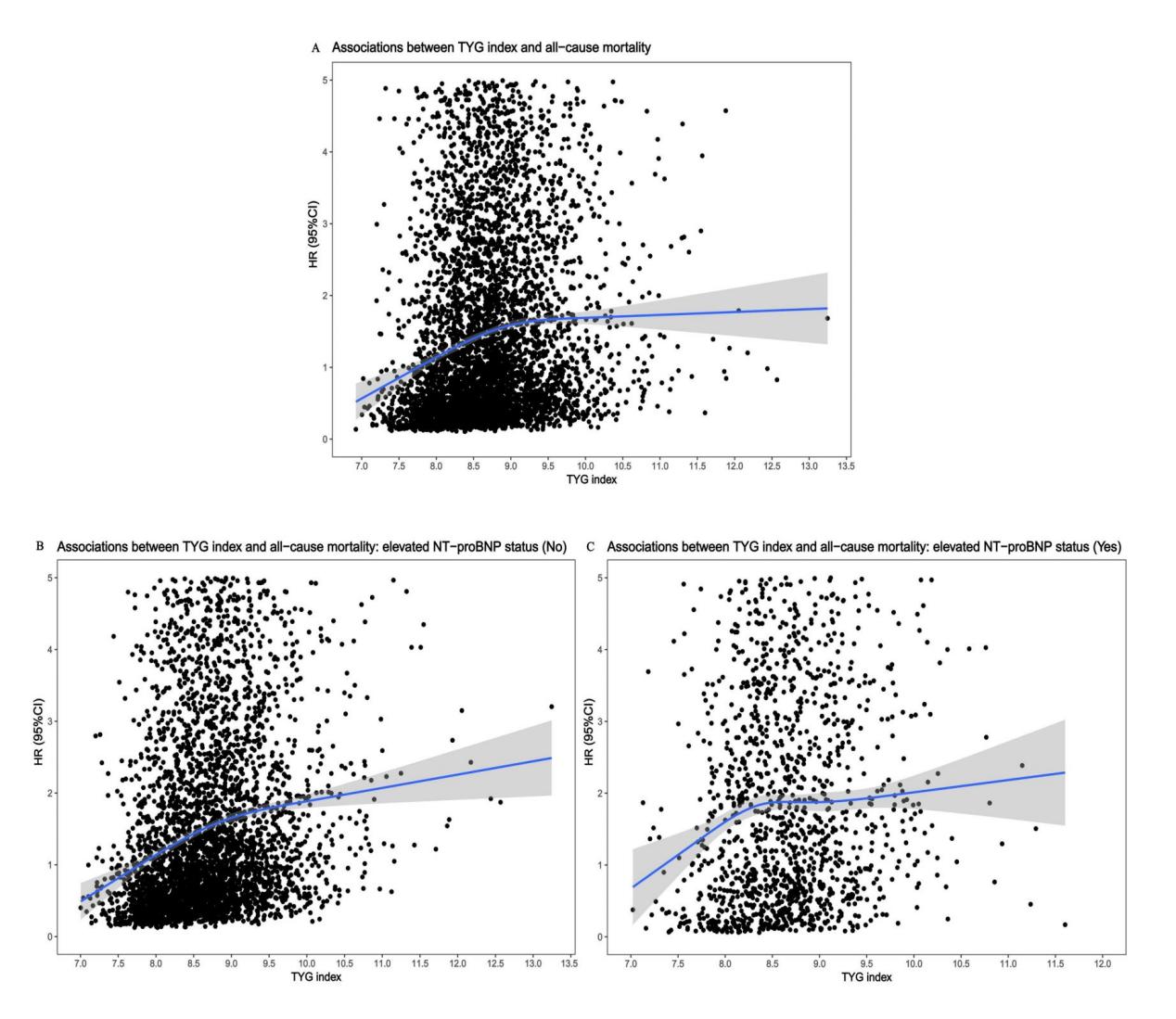


Figure 6

Figure S1: RCS models adjusted for age, gender, race, education level, BMI, hypertension, diabetes, smoking status, TC, HDL-C, urea, creatinine, UA, eGFR and use of hypoglycemic/lipid-lowering medications.

Figure S1A: total middle-aged and elderly individuals;

Figure S1B: middle-aged and elderly individuals without elevated NT-proBNP;

Figure S1C: middle-aged and elderly individuals with elevated NT-proBNP.

Figure S2-S3. Kaplan-Meier curves for TYG index and all-cause mortality in middle-aged and elderly individuals (study cohort 1).


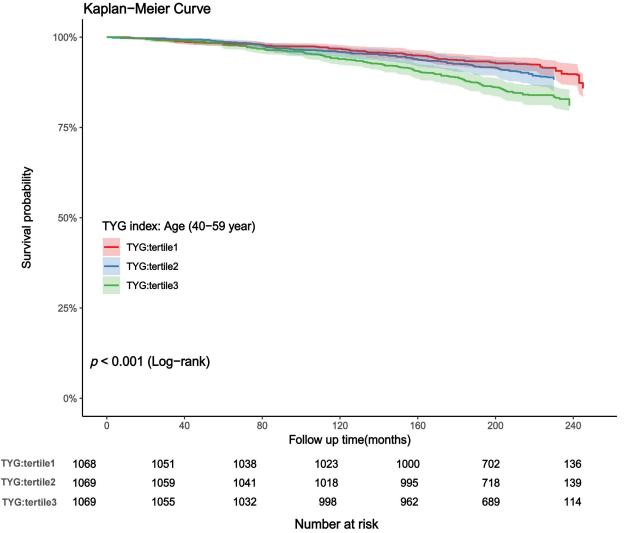

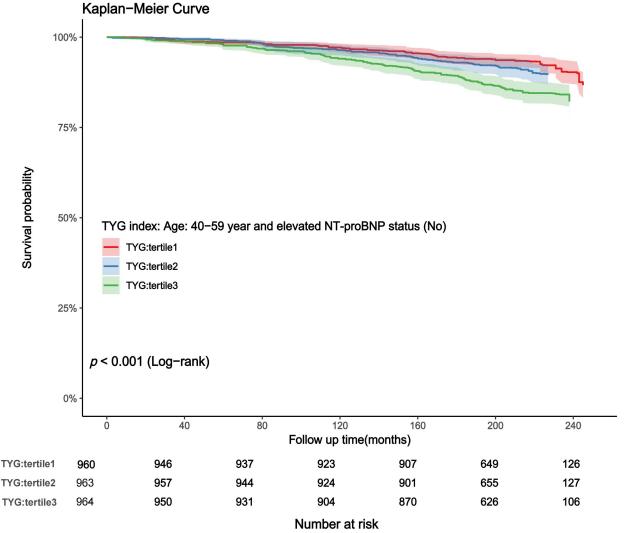


Figure S1 Figure S2

Figure S1: middle-aged and elderly individuals: Age 40-59 years;

Figure S2: middle-aged and elderly individuals: Age 40-59 years and without elevated NT-proBNP.

Figure S4-S5. RCS models demonstrating continuous associations of TYG index with all-cause mortality in middle-aged and elderly individuals (study cohort 1).


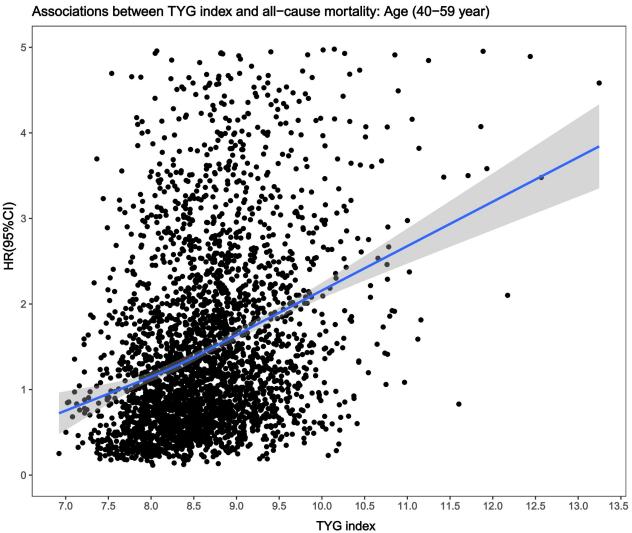

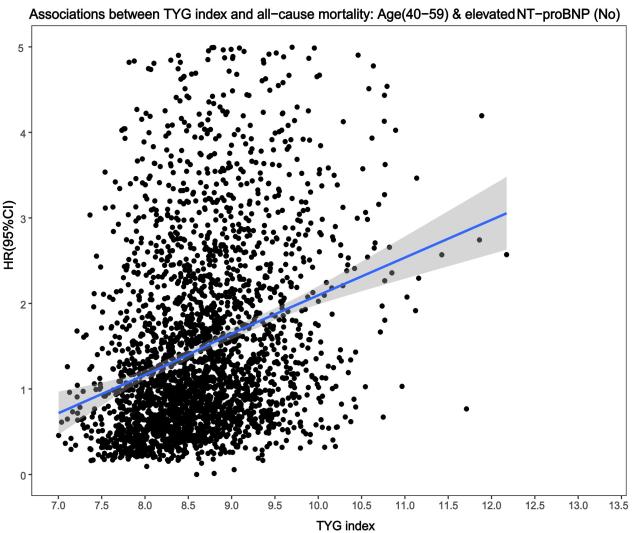


Figure S3 Figure S4

Figure S3/S4: RCS models adjusted for age, gender, race, education level, BMI, hypertension, diabetes, smoking status, TC, HDL-C, urea, creatinine, UA, eGFR and use of hypoglycemic/lipid-lowering medications.

Figure S3: middle-aged and elderly individuals: Age 40-59 years;

Figure S4: middle-aged and elderly individuals: Age 40-59 years and without elevated NT-proBNP.
